# Supplementary material for: Prediction of highly stable 2D carbon allotropes based on azulenoid kekulene
Source: Nat Commun. 2024 Mar 4;15:1953. doi: 10.1038/s41467-024-46279-8 (PMC10912223; doi:10.1038/s41467-024-46279-8)
Supplement: Supplementary file 1 — Supplementary Information [file 41467_2024_46279_MOESM1_ESM.pdf]

Supplementary Information for
Prediction of Highly Stable 2D Carbon Allotropes Based on Azulenoid Kekulene

Zhenzhe Zhang[a], Hanh D. M. Pham[a], Dmytro F. Perepichka[a,*], Rustam Z. Khaliullin[a,*]

[a] *Department of Chemistry, McGill University, 801 Sherbrooke St West, Montreal, H3A 0B8, Quebec, Canada*

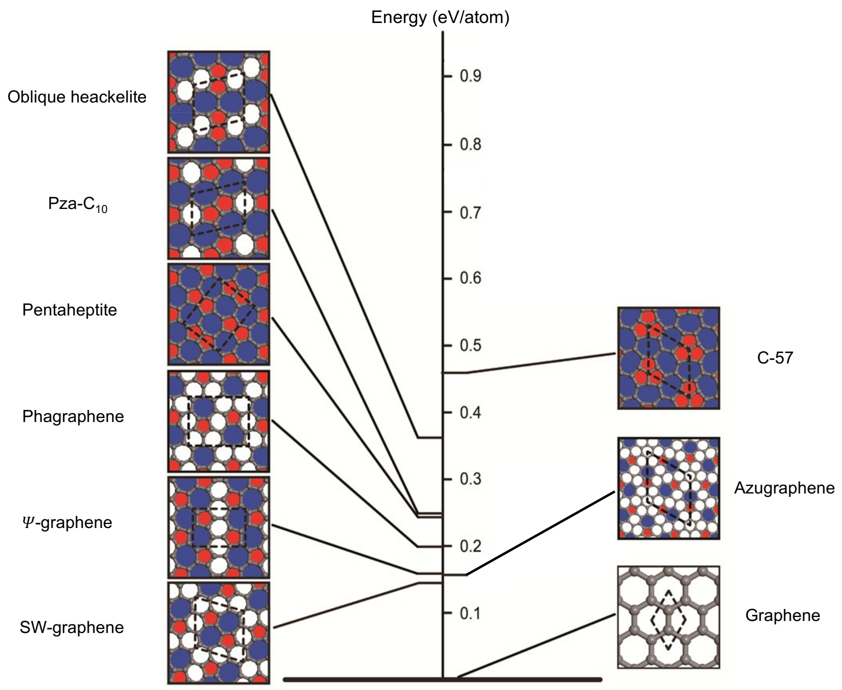

Supplementary Figure 1: **Structures of the previously reported 2D carbon allotropes containing azulene units.** SW-graphene [1], azugraphene [2], ψ-graphene [3], phagraphene [4], pentaheptite [5], Pza-C_{10} [6], oblique heackelite, and C-57 are shown. The energy per atom above graphene is shown. The dashed lines show unit cells. Adapted from Ref. [2] with permission from the Royal Society of Chemistry.

Supplementary Note 1. Stability of azulenoid kekulene

The cyclization strain energy of the azulenoid kekulene (AK) molecule was calculated by comparing its energy to that of its linear isomer (Supplementary Figure 2). Calculations were performed using Perdew-

*Corresponding author
Email addresses: dmytro.perepichka@mcgill.ca (Dmytro F. Perepichka), rustam.khaliullin@mcgill.ca (Rustam Z. Khaliullin)

Burke-Ernzerhof (PBE) exchange-correlation functional as described in Methods in the main text. For the AK molecule and linearized polymer, the integration over the Brillouin zone was performed using the $7 \times 7 \times 1$ and $7 \times 7 \times 1$ Monkhorst-Pack k-point meshes, respectively.

The calculations indicate that the AK macrocycle is $0.16\,\mathrm{eV}/\mathrm{azulene}$ unit more stable than its linearized isomer. This indicates that the strain energy of cyclization is fully compensated by the aromatic stabilization of the AK macrocycle. Thus, the low strain and aromaticity of AK explain the stability of the AK-based 2D polymers discussed in this work.

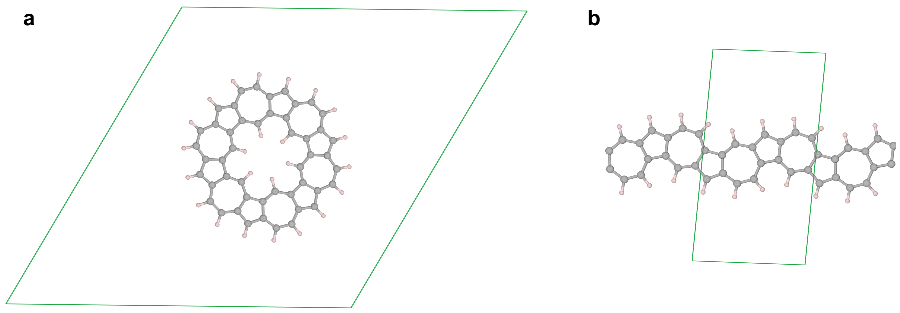

Supplementary Figure 2: **Structures of (a) the azulenoid kekulene macrocycle and (b) its linearized isomer.**. The green parallelogram shows the periodic box used in the calculations.

Supplementary Note 2. Alternative measure of stability of 2D carbon allotropes

In the main text, the stability of carbon allotropes is measured by comparing their total energy per atom to that of graphene. Although this measure is employed in all previous studies of carbon allotropes, it is important to consider its limitations for this study. If the separation between the AK units increased significantly, this measure of stability would decrease to zero, making it difficult to compare the stability of AK-based and non-AK carbon allotropes meaningfully. To make an illustrative example, AKC-$[100, 0]$ with well-separated AK units could be claimed, based on this measure, as the most stable carbon allotrope, which is meaningless because this material mostly consists of graphene.

This measure is also problematic because it makes it difficult to compare the stability among different AKCs. For example, it is unclear whether AKC-$[6, 0]$ is more stable than AKC-$[5, 1]$ because of the stronger interactions between the AK units or simply because of the larger distance between them.

In order to reduce the trivial stabilization effect due to the spatial separation between the AK units, we introduced an alternative measure of stability of azulene-based materials, AKCs and previously studied carbons. In this measure denoted E_{x}, the energy of the unit cell (E_{uc}) of an allotrope above the graphene is divided by the total number of non-benzoid atoms in the unit cell ($N_{\mathrm{nbz,uc}}$), not by the total number of

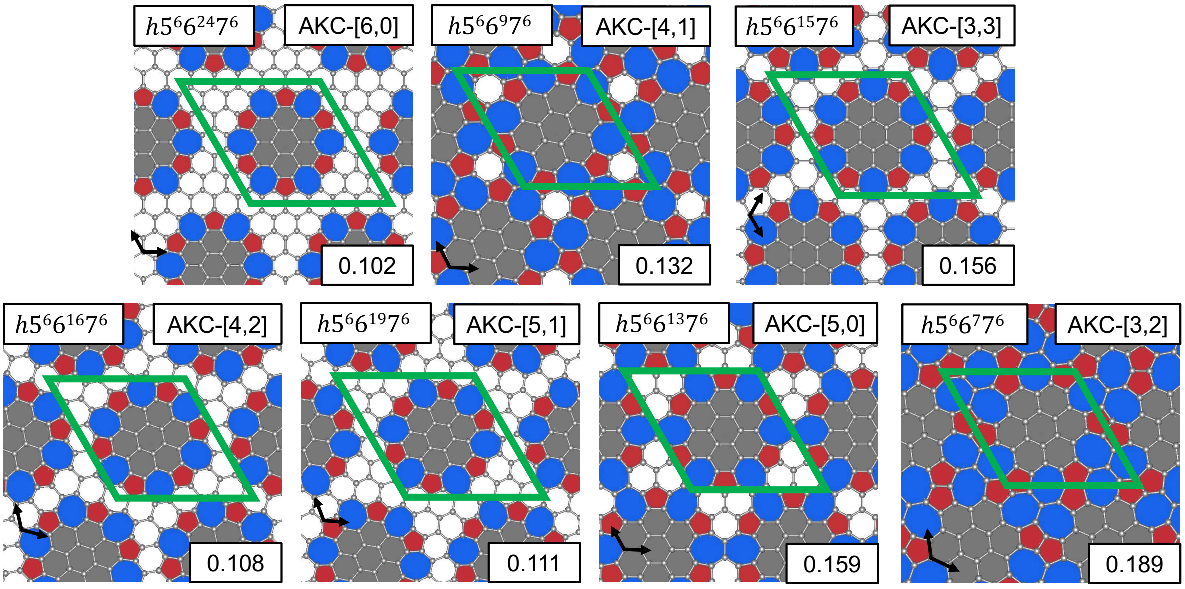

Supplementary Figure 3: **Alternative nomenclature for AKC materials.** The nomenclature proposed for 2D carbon allotropes [7] is shown in the upper left corner. According to another taxonomy (see Section 2 in Ref. [7]), AKC-$[n, m]$ material can be viewed as a defect in the graphene lattice and named $d5^66^77^6@h6^1$-$[(n, m); (-m, n-m)]$, where n and m refer to integers in the AKC-$[n, m]$ label.

atoms (N_{uc}):

$$E_x = \frac{E_{\mathrm{uc}} - N_{\mathrm{uc}} \times E_{\mathrm{graphene/atom}}}{N_{\mathrm{nbz,uc}}} = \frac{N_{\mathrm{uc}}}{N_{\mathrm{nbz,uc}}} \left(E_{\mathrm{allotrope/atom}} - E_{\mathrm{graphene/atom}}\right) \tag{1}$$

Non-benzoid atoms are those that are surrounded by hexagonal rings on all sides.

Supplementary Table 1 shows that, according to the new measure, AKC materials described in this work are still among the most stable 2D carbon allotropes. Another important observation enabled by E_x scale is that AKC-$[4, 2]$ stands out among the AKC structures as the material with the strongest interaction between its AK units. In this material, the pentagonal rings are directly bonded to the hexagonal rings of the neighboring AK units, making the material remarkably stable.

Supplementary Note 3. *Ab initio* molecular dynamics

Ab initio molecular dynamics (AIMD) calculations were performed for AKC-$[6, 0]$ and phagraphene to assess and compare the kinetic stability of these two materials at elevated temperatures. Supplementary Figure 4 shows that pentagonal and heptagonal rings in both phagraphene and AKC-$[6, 0]$ do not undergo a transformation into hexagonal rings when the systems were heated to 2500 K, despite significant distortions of all bonds at such high temperatures.

Material	Energy above graphene (eV/atom)	$N_{\mathbf{uc}}$	$N_{\mathbf{nbz}}$	$E_{\mathbf{x}}$ (eV/atom)
AKC-[4,2]	0.108	56	48	0.126
AKC-[5,1]	0.111	62	48	0.143
AKC-[6,0]	0.102	72	48	0.153
AKC-[4,1]	0.132	42	36	0.154
AKC-[3,3]	0.156	54	48	0.175
AKC-[5,0]	0.159	50	42	0.189
AKC-[3,2]	0.189	38	32	0.224
SW-graphene	0.141	16	16	0.141
ψ-graphene	0.158	10	10	0.158
Phagraphene	0.199	20	20	0.199
Azugraphene	0.157	38	30	0.199
Pentaheptite	0.243	16	16	0.243
Pza-C_{10}	0.249	10	10	0.249
Oblique heackelite	0.362	12	12	0.362
C-57 carbon	0.459	12	12	0.459

Supplementary Table 1: Stability of carbon allotropes according to the alternative measure E_x that accounts for the presence of carbon atoms in hexagonal environments.

It should be emphasized that while the AIMD simulations here indicate that there are no low-lying barriers separating the systems from more stable structures, they do not suggest that phagraphene and AKC-[6, 0] will remain stable at such high temperatures. In fact, the synthesized phagraphene nanoribbons are known to undergo transition at lower temperatures [8]. Substantial overestimation of phase transition temperatures in molecular dynanamics simulations is well known and can be explained by the very small size of simulated systems, limited duration of AIMD simulations (∼100 ps in our case), and the absence of defects in simulated systems. Since the AIMD-determined transition temperature cannot be directly compared to the experimental temperature, the AIMD simulations were stopped at the arbitrarily high temperature without observing the transition or collapse of the AK-based structure.

The AIMD simulations were performed for AKC-[6, 0] and phagraphene with simulation cells of comparable size: 72 atoms in the AKC-[6, 0] cell and 80 atoms in the phagraphene cell. The time step of 2 fs was used in all simulations. A 12 ps constant volume constant temperature (NVT) simulation with the Nosé-Hoover thermostat [9], was performed to equilibrate the systems at 300 K. Subsequently, a 125 ps NVT simulation was carried out for AKC-[6, 0] with the temperature gradually increasing from 300 K to 3000 K. Similarly, a 90 ps NVT simulation was carried with the temperature rising from 300 K to 2500 K. Additional 23 ps constant pressure (NPT) simulations were started for both systems from NVT snapshots at 1000 K and the

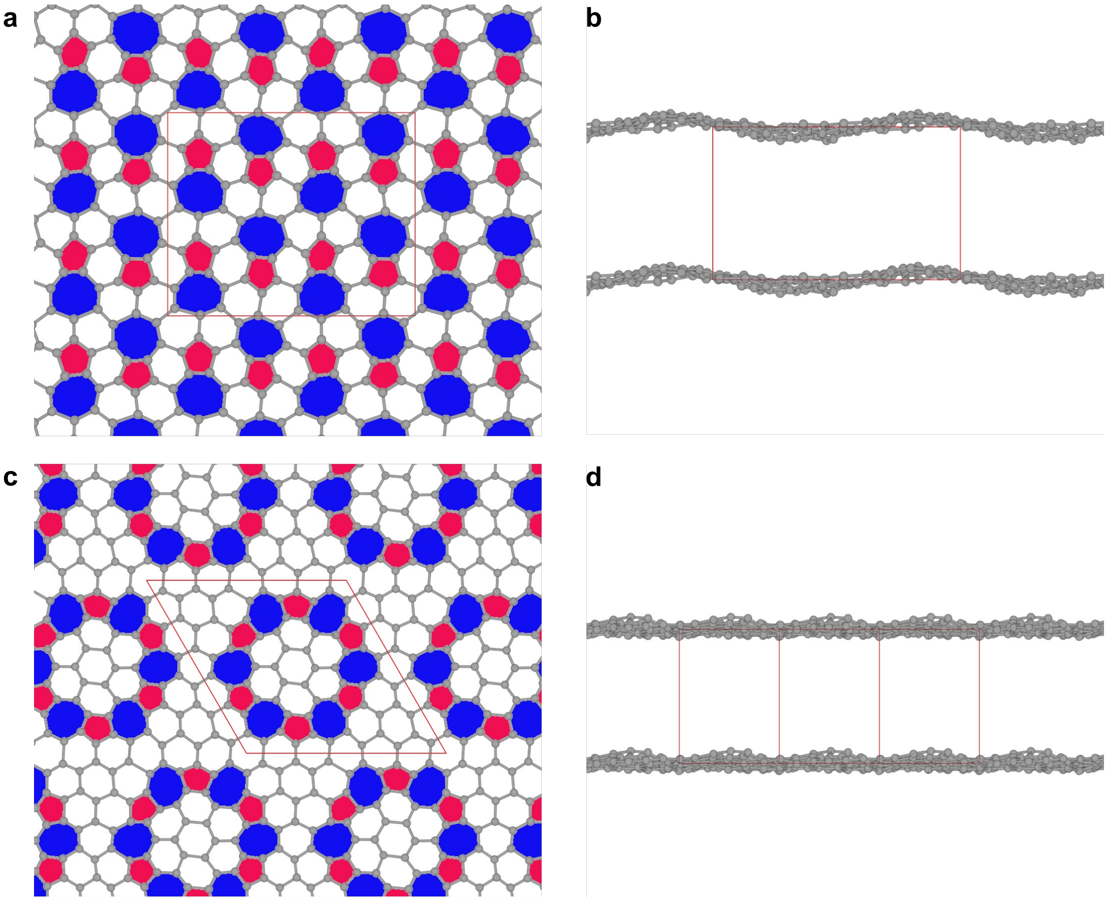

Supplementary Figure 4: **AIMD snapshots of phagraphene at 2500 K and AKC-**$[6,0]$ **3000 K.** The top and side views of phagraphene are shown in **a** and **b**, respectively. The top and side views of AKC-$[6,0]$ are shown in **c** and **d**, respectively.

temperature was gradually increased to 2500 K. The temperature in NVT simulations was maintained using the Nosé-Hoover thermostat with the velocities scaled each 50 steps. The NPT simulation was controlled by the Langevin thermostat [10] with the friction coefficient of atoms and lattice degrees of freedom at $10\,\mathrm{ps}^{-1}$.

Supplementary Note 4. Parity of nodes of AK-based structures

As stated in the main text, the electronic structure of the proposed materials is to a large extent determined by the relative position of AK units in the lattice. All $[6,0]$, $[3,3]$ and $[4,1]$ materials are semiconductors, while the others – $[4,2]$, $[5,1]$, $[5,0]$ and $[3,2]$ – are metals or semimetals.

We observe that semiconductors are obtained when the nodes of the underlying porous graphene matrix are *even*, whereas semimetals or metals are obtained when the nodes are *odd*. A node of the porous graphene matrix is defined as a group of atoms forming connections to the three identical neighbor nodes. The nodes are shown with white color in Supplementary Figure 3. A node is called *even* if there is a hexagon of carbon

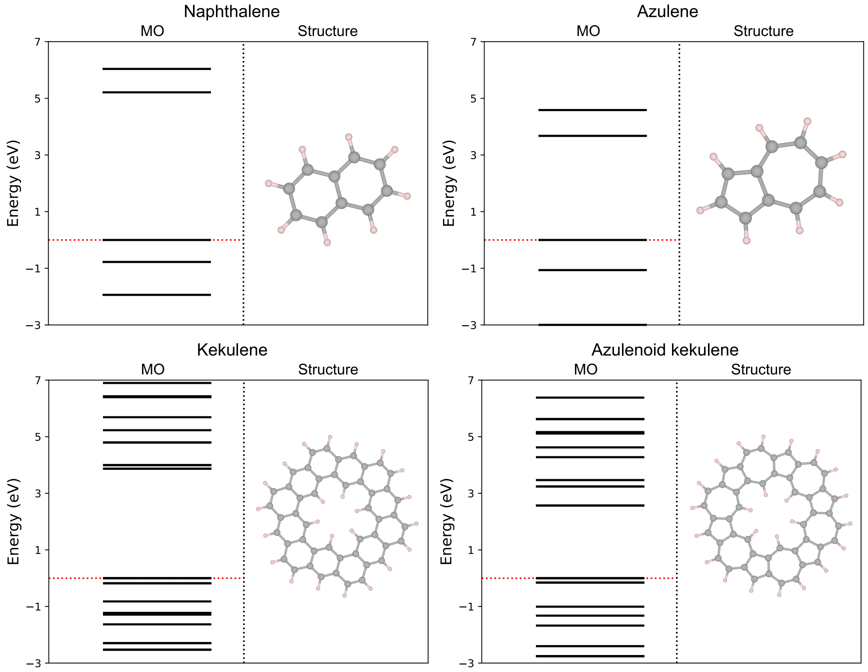

Supplementary Figure 5: **PBE0/6-31G(d) molecular orbital diagrams for naphthalene, azulene, kekulene and azulenoid kekulene molecules.** HOMO energies are used as the zero-energy references (dashed line).

atoms in its center. Examples of even nodes include nodes of [6, 0], [3, 3] and [4, 1] materials. A node is called *odd* if there is a single carbon atom in its center. Materials [4, 2], [5, 1], [5, 0] and [3, 2] contain odd nodes. Interestingly, even nodes represent closed-shell molecules with paired electrons (e.g. benzene, triphenylene, coronene), whereas odd nodes represent open-shell radicals (e.g. triangulenes).

Supplementary Note 5. Molecular orbital diagrams for molecular building blocks

Computational modeling of molecular structures was performed using density functional theory as implemented in Gaussian 16, Revision A.03 [11]. The optimization of molecular geometries and calculation of molecular orbital diagrams were carried out using PBE0 hybrid exchange-correlation functional [12, 13] in combination with the 6-31G(d) basis set. Molecular orbital diagrams for naphthalene, azulene, kekulene and azulenoid kekulene molecules are shown in Supplementary Figure 5.

The depicted molecular orbital energies elucidate that the HOMO-LUMO gap for azulene is 1.54 eV narrower than that of naphthalene. Similarly, the unoccupied molecular orbitals of azulenoid kekulene also lie lower than those of kekulene.

Supplementary Table 2: Comparison of electronic structure properties of 2D π-conjugated materials computed with an interlayer distance of 10 Å and 20 Å. Within each family, materials are listed in order of increasing energy.

Material	PBE energy above hull (20 Å), eV/atom	PBE energy above hull (10 Å), eV/atom	HSE bandgap (secondary gap), eV (20 Å)	HSE bandgap (secondary gap), eV (10 Å)
AKC-[6, 0]	0.1554	0.1550	0.833	0.831
AKC-[4, 2]	0.1612	0.1609	DC*	DC*
AKC-[5, 1]	0.1639	0.1636	DC*	DC*
AKC-[4, 1]	0.1855	0.1851	0.435	0.433
AKC-[3, 3]	0.2090	0.2086	0.542 (0.801)	0.540 (0.798)
AKC-[5, 0]	0.2122	0.2118	M**	M**
AKC-[3, 2]	0.2419	0.2416	M**	M**
PAK-[6, 0]	0.1857	0.1854	0.112	0.109
PAK-[5, 1]	0.1941	0.1943	DC*	DC*
PAK-[4, 2]	0.1992	0.1989	DC*	DC*
PAK-[4, 1]	0.2305	0.2302	0.280	0.277
PAK-[3, 3]	0.2452	0.2449	0.362 (0.562)	0.360 (0.561)
PAK-[5, 0]	0.2509	0.2507	DC*	DC*
PAK-[3, 2]	0.2915	0.2912	DC*	DC*
PG-[6, 0]	0.1423	0.1422	1.729	1.728
PG-[5, 1]	0.1702	0.1701	DC*	DC*
PG-[3, 3]	0.1765	0.1763	2.181	2.180
PG-[4, 2]	0.1845	0.1844	DC*	DC*
PG-[5, 0]	0.2117	0.2116	DC*	DC*
PG-[4, 1]	0.2129	0.2129	3.178	3.178
PG-[3, 2]	0.2387	0.2387	DC*	DC*

DC*- semimetal with Dirac cones; M**- metal with zero bandgap.

Supplementary Note 6. Effect of the interlayer distance

The choice for the interlayer distance of 10 Å was confirmed by recalculating the energy and band gaps using 20 Å interlayer distance. Comparison of the results for the two models (Supplementary Table 2) shows the discrepancy in both energy and band gaps is negligible, with the maximum difference of 0.0004 eV/atom and 0.003 eV, respectively. The band structure diagrams for the two models are nearly identical (Supplementary Figure 7). The small differences confirm the validity of the model with the 10 Å interlayer distance.

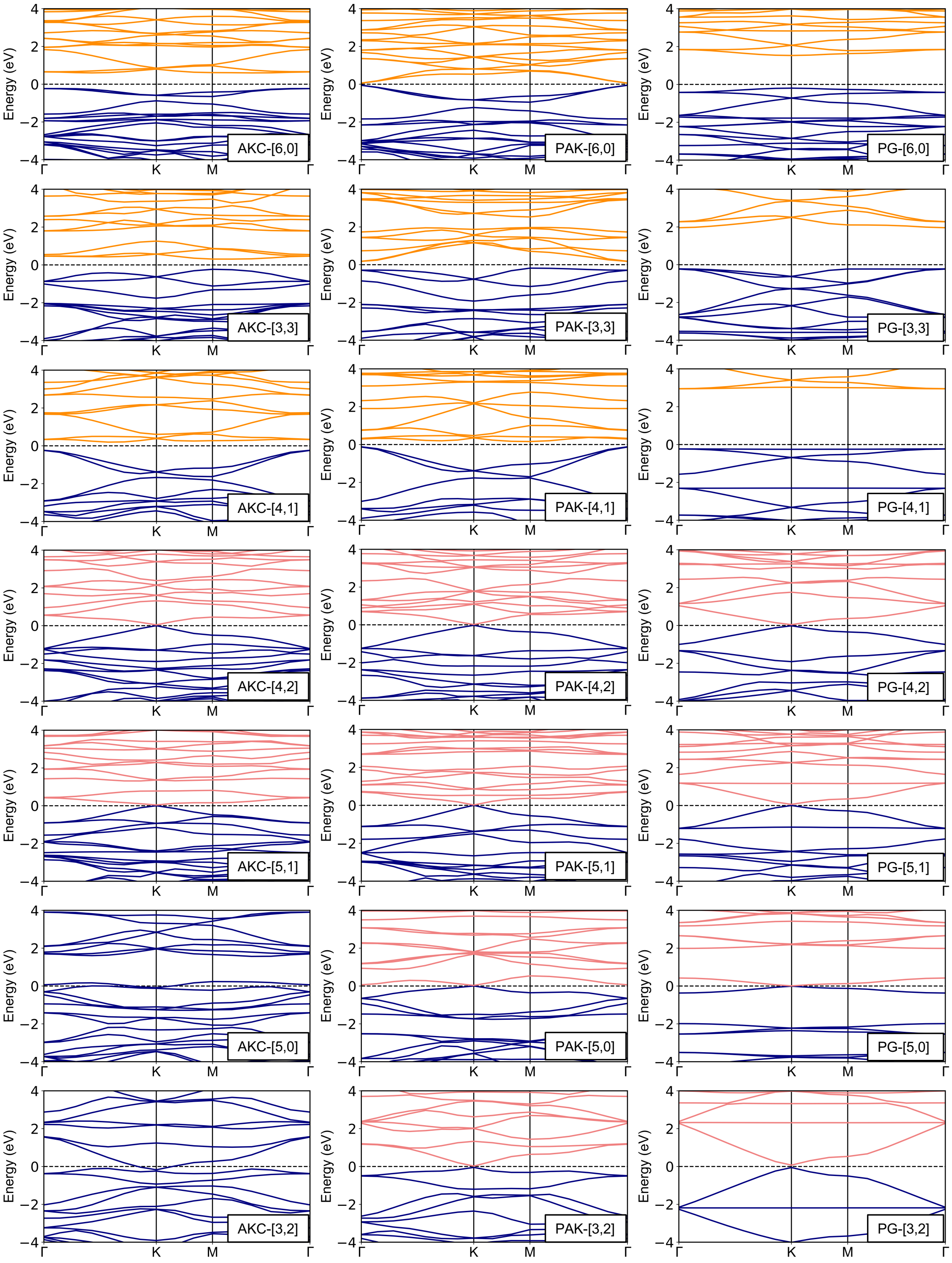

Supplementary Figure 6: **HSE band structure diagrams for seven materials from the AKC, PAK, and PG families.** The orange color denotes conduction bands of semiconductors, the red color denotes the conduction bands of Dirac-cone semimetals, and the blue color denotes the conduction bands of metals and valence bands of all materials.

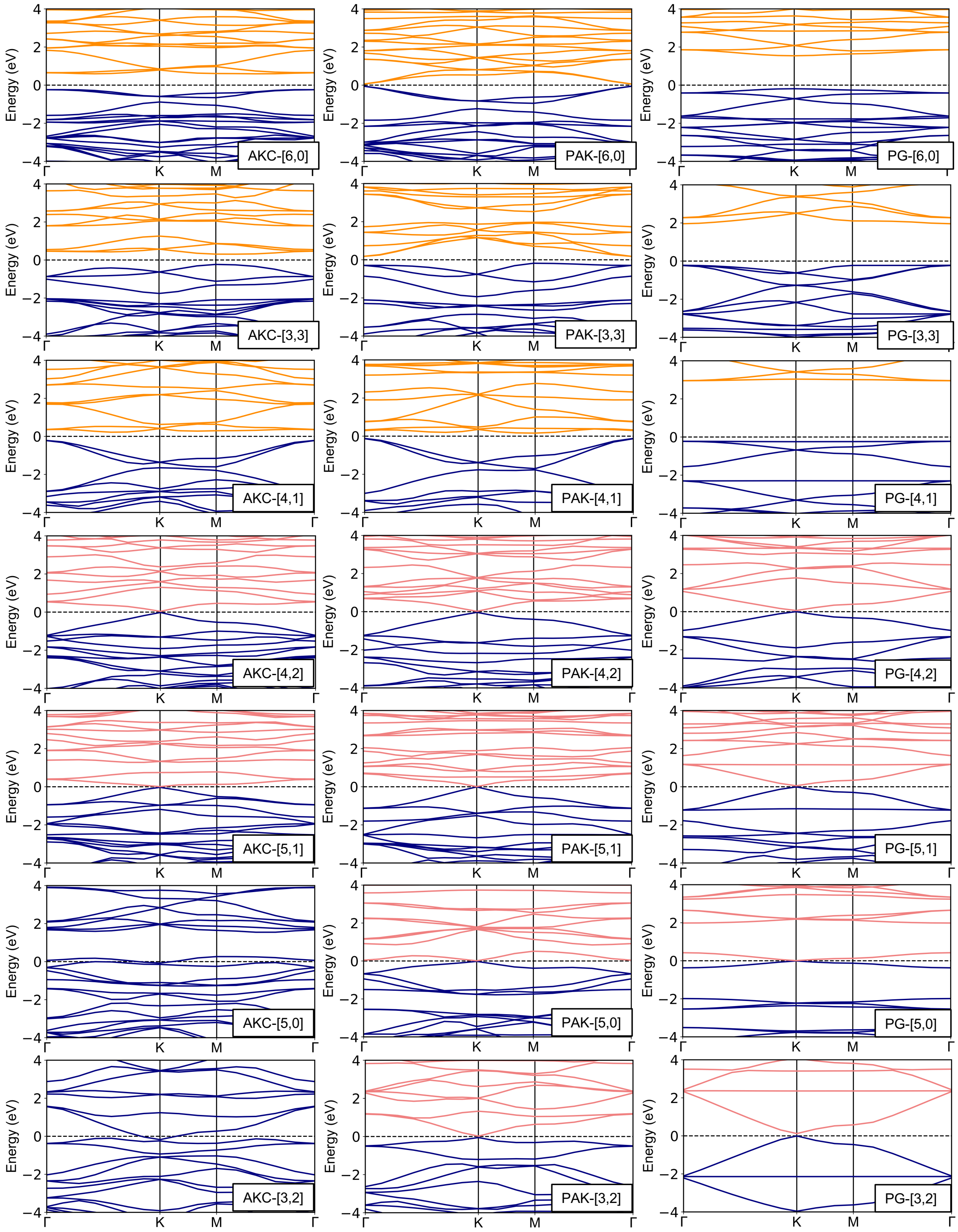

Supplementary Figure 7: **HSE band structure diagrams for materials from the AKC, PAK, and PG families computed for the** 20 Å **interlayer distance.** The color coding is the same as in Supplementary Figure 6.

Supplementary references

[1] Yin, H. *et al.* Stone-Wales graphene: a two-dimensional carbon semimetal with magic stability. *Phys. Rev. B* **99**, 041405 (2019).

[2] Liu, J. & Lu, H. Azugraphene: a new graphene-like hexagonal carbon allotrope with dirac cones. *RSC Adv.* **9**, 34481–34485 (2019).

[3] Li, X., Wang, Q. & Jena, P. ψ-graphene: a new metallic allotrope of planar carbon with potential applications as anode materials for lithium-ion batteries. *J. Phys. Chem. Lett.* **8**, 3234–3241 (2017).

[4] Wang, Z. *et al.* Phagraphene: a low-energy graphene allotrope composed of 5–6–7 carbon rings with distorted dirac cones. *Nano Lett.* **15**, 6182–6186 (2015).

[5] Molepo, M. P., Mapasha, R. E., Obodo, K. O. & Chetty, N. First principles calculations of pentaheptite graphene and boronitrene derivatives. *Comput. Mater. Sci.* **92**, 395–400 (2014).

[6] Luo, X. *et al.* Two-dimensional superlattice: modulation of band gaps in graphene-based monolayer carbon superlattices. *J. Phys. Chem. Lett.* **3**, 3373–3378 (2012).

[7] Girão, E. C., Macmillan, A. & Meunier, V. Classification of sp2-bonded carbon allotropes in two dimensions. *Carbon* **203**, 611–619 (2023).

[8] Fan, Q. *et al.* Nanoribbons with nonalternant topology from fusion of polyazulene: carbon allotropes beyond graphene. *J. Am. Chem. Soc.* **141**, 17713–17720 (2019).

[9] Nosé, S. A unified formulation of the constant temperature molecular dynamics methods. *J. Chem. Phys.* **81**, 511–519 (1984).

[10] Hoover, W. G., Ladd, A. J. & Moran, B. High-strain-rate plastic flow studied via nonequilibrium molecular dynamics. *Phys. Rev. Lett.* **48**, 1818 (1982).

[11] Frisch, M. J. *et al.* Gaussian˜16 Revision A.03 (2016). Gaussian Inc. Wallingford CT.

[12] Adamo, C. & Barone, V. Toward reliable density functional methods without adjustable parameters: the PBE0 model. *J. Chem. Phys.* **110**, 6158–6170 (1999).

[13] Perdew, J. P., Burke, K. & Ernzerhof, M. Generalized gradient approximation made simple. *Phys. Rev. Lett.* **77**, 3865 (1996).
